# Transfusion of packed red blood cells in adults with sickle cell anemia treated at an emergency hospital

**DOI:** 10.1590/1806-9282.20230816

**Published:** 2024-02-26

**Authors:** Bianca Cansian, João Carlos Pina Faria, Roseli Oselka Saccardo Sarni

**Affiliations:** 1 ABC Faculty of Medicine, University Center - Santo André (SP), Brazil.; 2 Universidade Municipal de São Caetano do Sul, Universidade Nove de Julho, ABC Faculty of Medicine, University Center - São Caetano do Sul (SP), Brazil.; 3 Universidade Federal de São Paulo, ABC Faculty of Medicine, University Center - São Paulo (SP), Brazil.

**Keywords:** Transfusion medicine, Erythrocyte transfusion, Sickle cell, Emergency treatment, Prescriptions

## Abstract

**OBJECTIVE:**

The aim of this study was to analyze the prescription of packed red blood cells performed by emergency physicians for adults with sickle cell anemia.

**METHODS:**

Transfusions performed in adults with sickle cell anemia treated at an emergency service in São Bernardo do Campo, São Paulo Brazil, between January 2018 and January 2022 were evaluated. For data comparison, the chi-square^2^ test was used. The significance level adopted was 5%.

**RESULTS:**

A total of 114 transfusions were performed. The mean age was 41.8±16.4 years, and pretransfusion hemoglobin was 6.1±1.23 g/dL. Regarding the indication, the adequacy of transfusions performed in symptomatic individuals was significantly higher compared to asymptomatic individuals (100% vs. 3.9%, p<0.001). Symptomatic individuals received excessive volumes of packed red blood cells less frequently than asymptomatic individuals (17.5% vs. 56.9%, p<0.001). The filtered subtype, indicated for sickle cell anemia, was prescribed in only a quarter of the patients. However, non-indicated subtypes were frequently prescribed.

**CONCLUSION:**

This study found low adequacy in the indication and calculation of the transfusion volume of packed red blood cells in asymptomatic individuals. Few patients received filtered red blood cells, resulting in increased risks of transfusion reactions. On the contrary, non-indicated subtypes were prescribed in a quarter of transfusions, which resulted in higher costs and delay in receiving packed red blood cells.

## INTRODUCTION

SCA is the most prevalent hereditary hematological disorder in the world^1^, affecting millions of individuals^2^. In Brazil, according to the National Neonatal Screening Program of the Ministry of Health, in 2019, 1,214 cases of sickle cell disease and 61,021 with sickle cell trait were diagnosed^3^.

The disease is caused by a mutation in the hemoglobin β gene (Hb). The homozygous form is called SCA and represents the most severe form^4^. The heterozygous form (sickle cell trait) is usually asymptomatic and is present in more than 2 million Brazilians^5^.

The average survival of individuals with SCA has increased in recent decades, but it still remains below the general world average. In the United States, life expectancy increased from 28 to 43 years between 1979 and 2017^6^. In Brazil, life expectancy is 29.4 years for men and 33.3 years for women^7^.

The treatment of acute complications of SCA depends on age, type of complication, and severity^8^. Concentrated RBC transfusion is used in some situations, especially when hemodynamic decompensation occurs^9^ ^,^ ^10^ ^,^ ^11^.

Attention is needed when prescribing RBC to individuals with SCA. As these are patients with chronic anemia, the transfusion trigger in acute complications should be based mainly on the clinical picture and not on the Hb value. The volume should preferably be one RBC at a time, except in situations of hemodynamic decompensation and severe bleeding. RCB should be filtered when collecting blood or at the time of transfusion to reduce the infusion of neutrophils to the receiver. These neutrophils are responsible for the nonhemolytic febrile reaction, the main reason for fever during transfusion in multiple patients. The occurrence of this reaction leads to the interruption of the transfusion and the request for a new blood component, delaying the procedure and increasing the costs and risks of transfusion reactions^12^.

The objective of this study was to analyze the prescription of packed RBCs for adults with SCA performed by emergency physicians.

## METHODS

This is a cross-sectional study, based on data collection from hemotherapy procedure request forms. The study was carried out at the Hospital de Urgências de São Bernardo do Campo, São Paulo Brazil, a tertiary public hospital equipped with a transfusion agency supervised by a hematologist responsible for the facility, available for consultation by other hospital physicians in cases of transfusion doubt. The hospital provides free public healthcare services for a region with approximately 850,000 inhabitants.

Records of patients with SCA aged over 18 years, who received RBC transfusion prescribed by emergency physicians from the medical clinic, from January 2018 to January 2022 were included. Forms with incomplete data and prophylactic transfusions (indicated for the prevention of stroke or surgical preparation) were excluded.

To assess the adequacy of the RBC prescription, three parameters were considered: indication for transfusion, considering the patient’s clinical condition and the hemoglobin (Hb) value; prescribed volume (number of prescribed RBC bags); and choice of RBC subtype (considering the filtrate as an indicated subtype and the irradiated and washed subtypes as non-indicated). To assess the adequacy, the recommendations of the Ministry of Health of Brazil, from 2015, were used^13^.

Qualitative variables were presented as absolute numbers and percentages. For the analysis of qualitative data, the chi-square test was used. Poisson regression analysis with robust variance was performed to estimate the prevalence ratio, considering adequate as the outcome variable. Data were analyzed using the Stata version 14.0 statistical package, and the significance level adopted was 5%.

The study was approved by the Research Ethics Committee of Centro Universitário FMABC (opinion number: 3.286.784), CAAE 11199319.2.0000.0082.

## RESULTS

Table 1 presents the general characteristics of the studied population. A total of 114 transfusions were performed during the study period in individuals with SCA, of whom 55.3% were females. The mean age was 41.8±16.4 years, pretransfusion Hb was 6.1±1.23 g/dL, and 55.3% had some symptoms of acute complication of SCA before the transfusion. Among the symptomatic patients, individuals with hemodynamic decompensation, sepsis, and acute chest syndrome were transfused. Half of the transfusions were indicated based on the Hb values between 5 and 7 g/dL. Six out of ten received two or more RBC grants. In 41.2% of the transfusions, one or more subtypes of RBC were prescribed. Filtered RBC was prescribed more.

**Table 1.** Characterization of the studied sample (n=114).

Variable	n	%
Sex		
Male	51	44.7
Female	63	55.3
Age (years)		
Median 41.8±16.4		
18-30	28	24.6
30-60	68	59.6
≥60	18	15.8
Clinical condition		
Asymptomatic	51	44.7
Symptomatic	63	55.3
Hemodynamic decompensation	41	36
Sepsis	14	12.3
Acute chest syndrome	8	7
Pretransfusional hemoglobin (g/dL)		
Median 6.1±1.23		
<5 g/dL	24	21.1
5-7 g/dL	57	50
≥7 g/dL	33	28.9
Volume		
1 RBC bag	45	39.5
2 RBC bags	59	51.7
3 RBC bags	10	8.8
RBC subtypes		
Not prescribed	67	58.8
Prescribed	47	41.2
Filtered	31	27.2
Irradiated	25	21.9
Washed	5	4.4

RBC: concentrated RBC.

Adequacy in relation to the transfusion trigger (pretransfusion Hb), prescribed volume, and choice of subtypes (filtered, irradiated, and washed) is shown in Table 2. Adequacy with a transfusional trigger of Hb<5 g/dL, between 5 and 7 g/dL, and ≥7 g/dL was 100%, 64.9%, and 6.1%, respectively, and the adequacy of the prescribed volume was 100% in prescriptions with one bag of RBC and 49.2% with two bags of RBC. No prescription with more than two bags of RBC was in accordance with the recommendations of the Ministry of Health. In the assessment of prescribed subtypes, filtered RBC prescriptions were considered adequate. There was no indication for irradiated and washed RBC prescriptions.

**Table 2.** Analysis of the adequacy of the prescription of packed red blood cells performed by emergency physicians of a public hospital.

Variable	Adequate	
	n	%
Hemoglobin		
<5 g/dL	24	100
5-7 g/dL	37	64.9
≥7 g/dL	2	6.1
Volume		
1 RBC bag	45	100
2 RBC bags	29	49.2
RBC subtypes		
Filtered	31	100
Irradiated/washed	0	0

RBC: concentrated RBC.

The indication for transfusion (considering the clinical status and pretransfusion Hb) was correct in all symptomatic patients and in only 3.9% of asymptomatic patients (p<0.001). The prescribed volume was adequate in 82.5% of symptomatic individuals and in 43.1% of asymptomatic ones (p<0.001). In the analysis of the prescribed subtypes, adequacy was 15.9% in symptomatic patients and 21.6% in asymptomatic patients, and there was no statistically significant difference (p=0.43) (Table 3).

**Table 3.** Comparison of adequacy of red blood cell prescription by emergency physicians in an emergency department for asymptomatic and symptomatic patients with sickle cell disease.

Variable	Adequate		Inadequate		PR	95%CI	p-value
	n	%	n	%			
Indication							
Asymptomatic	2	3.9	49	96.1	1	6.5-99.8	<0.001*
Symptomatic	63	100	0	0	25.5		
RBC volume							
Asymptomatic	22	43.1	29	56.9	1	1.4-2.7	<0.001*
Symptomatic	52	82.5	11	17.5	1.9		
RBC subtypes							
Asymptomatic	11	21.6	40	78.4	1	0.3-1.6	0.43
Symptomatic	10	15.9	53	84.1	0.7		

*P: significance level of the chi-square test. RP: prevalence ratio; CI: confidence interval; RBC: concentrated RBC.

## DISCUSSION

The present study showed greater adequacy of the prescription of RBC transfusions performed by emergency physicians in terms of indication and volume for symptomatic individuals with SCA at the time of prescription compared to asymptomatic patients.

The prescription of RBC without appropriate indications in asymptomatic individuals increases transfusion risks, including future transfusions through alloimmunization, a frequent complication in polytransfused patients. The purpose of RBC transfusion in patients with SCA is not to normalize Hb values, but to improve oxygen transportation by increasing the healthy Hb count aided by the hemodilution of the altered Hb^7^ ^,^ ^11^. Individuals with SCA have baseline Hb concentrations below 10 g/dL; therefore, these individuals are adapted to a chronic condition of anemia^14^. The main recommendations for RBC transfusion for patients with SCA are as follows: a decrease in Hb ≥2 g/dL compared to baseline values and/or the presence of an acute complication such as a vaso-occlusive refractory crisis to conventional treatment, acute chest syndrome, splenic or hepatic sequestration, transitory aplastic crisis, or hemodynamic decompensation^10^ ^,^ ^12^. RBC transfusion for asymptomatic patients should be performed in restricted situations such as preoperative preparation and prophylaxis for primary and secondary ischemic stroke^15^ ^,^ ^16^ ^,^ ^17^.

The RBC-infused volume was considered excessive in half of the prescriptions with two bags and in all prescriptions with three. High RBC-infused volumes can cause blood hyperviscosity, increasing the risk of ischemic stroke, acute chest syndrome, and circulatory overload, especially in the elderly^10^. To minimize risks, posttransfusion Hb should not exceed 11 g/dL^18^. In adults, the transfusion of an RBC bag raises Hb by approximately 1 g/dL, and this reference can be used to calculate the number of RBC bags needed^13^. Some individuals may benefit from the use of diuretics to avoid circulatory overload, but this conduct should not be routinely performed^19^ ^,^ ^20^.

The RBC-filtered subtype is indicated to reduce the risk of a nonhemolytic febrile reaction caused by donor neutrophils, but only a quarter of individuals received this subtype. The development of this transfusion reaction results in the necessity of interrupting the procedure to analyze the reason for the temperature rise and request another RBC, thus increasing costs and time for treatment and reducing RBC stocks^13^.

In SCA, there is no indication of irradiated and/or washed RBC. Irradiation is mainly indicated for severe immunodeficiencies or related donation. Washed RBC is indicated for patients who have already had severe allergic reactions related to the transfusion or who have a protein deficiency such as IgA^13^. These subtypes were unnecessarily prescribed to one in four adults with SCA, increasing treatment costs and delaying transfusion^21^.

The selection of phenotyped RBC can prevent alloimmunization, but it is not routinely performed in most emergency services in our country due to the time required for phenotyping^22^. RBC alloimmunization rates are significantly higher in individuals with SCA (18 to 47%) when compared to other diseases that are present with frequent transfusions. In addition, the risk of alloimmunization in SCA is up to 40 times greater when the transfusion is performed during the treatment of acute complications (such as those evaluated in this study) when compared to the group that receives chronic transfusions^23^. Improving the prescription of RBC concentrates can reduce the risks of transfusion reactions and contribute to the reduction of morbidity and mortality in individuals with SCA. Furthermore, it would lead to cost reduction for the hospital for the following reasons: there would be fewer transfusion prescriptions; even when indicated, a smaller volume (units) would be prescribed per transfusion; and there would be fewer unnecessary subtype prescriptions.

In a study carried out in the United States, 183 physicians working in hospitals answered a standardized questionnaire on transfusion practices (BEST-TEST exam). The average score was 51% correct (range: 20-85%). Approximately 40% received an hour or less of transfusion training, and 80% of participants reported that extra training in transfusion medicine would be helpful^24^. A Brazilian study applied the same questionnaire to 106 resident physicians from eight specialties in four different hospitals. The average number of correct answers was 43.5% (range: 15-80%). Most residents (73%) did not receive training in transfusion medicine during education or residency, and 93% would like to receive additional training. There was a clear deficit in the knowledge of the subject, indicating the need for change in the teaching of this area^25^. We did not find studies in the literature that specifically evaluated knowledge or transfusion suitability for individuals with SCA. As it has particularities with different indications from healthy individuals, medical knowledge about transfusion practices in individuals with SCA may be even lower than that described in previous studies. The data presented demonstrate the need for a review of the teaching plan of medical graduation institutions, adding the subject to the academic curriculum.

This study carried out the analysis of cases of acute decompensation in adults with SCA, treated at an emergency service, which makes the sample more homogeneous. As a limitation, we can mention the evaluation of patients from a single hospital. It is not possible to extrapolate the study results to other hospitals. However, the study site has a similar structure to other tertiary public hospitals in the country; therefore, there is a possibility that other hospitals may face similar issues in the prescription of RBC concentrates for individuals with SCA.

## CONCLUSION

The study observed low adequacy in the indication and calculation of the transfusion volume of packed RBCs in asymptomatic individuals. Few patients received filtered RBCs, resulting in increased risks of transfusion reactions. On the contrary, non-indicated subtypes were prescribed in a quarter of transfusions, which resulted in higher costs and delay in receiving the packed RBCs.

## References

[1] Wastnedge E, Waters D, Patel S, Morrison K, Goh MY, Adeloye D (2018). The global burden of sickle cell disease in children under five years of age: a systematic review and meta-analysis. J Glob Health.

[2] Ware RE, Montalembert M, Tshilolo L, Abboud MR (2017). Sickle cell disease. Lancet.

[3] Ministério da Saúde do Brasil (2023). Ministério da Saúde reforça a importância da detecção da doença falciforme.

[4] Piccin A, Murphy C, Eakins E, Rondinelli MB, Daves M, Vecchiato C (2019). Insight into the complex pathophysiology of sickle cell anaemia and possible treatment. Eur J Haematol.

[5] Loureiro MM, Rozenfeld S (2005). [Epidemiology of sickle cell disease hospital admissions in Brazil]. Rev Saude Publica.

[6] Payne AB, Mehal JM, Chapman C, Haberling DL, Richardson LC, Bean CJ (2020). Trends in sickle cell disease-related mortality in the United States, 1979 to 2017. Ann Emerg Med.

[7] Santo AH (2022). Sickle cell disease related mortality in Brazil, 2000-2018. Hematol Transfus Cell Ther.

[8] Neumayr LD, Hoppe CC, Brown C (2019). Sickle cell disease: current treatment and emerging therapies. Am J Manag Care.

[9] Ferreira FA, Benites BD, Costa FF, Gilli S, Olalla-Saad ST (2019). Recombinant erythropoietin as alternative to red cell transfusion in sickle cell disease. Vox Sang.

[10] Swerdlow PS (2006). Red cell exchange in sickle cell disease. Hematology Am Soc Hematol Educ Program.

[11] Rees DC, Robinson S, Howard J (2018). How I manage red cell transfusions in patients with sickle cell disease. Br J Haematol.

[12] Trompeter S, Massey E, Robinson S, Transfusion Task Force of the British Society of Haematology Guidelines Committee (2020). Position paper on international collaboration for transfusion medicine (ICTM) guideline ‘red blood cell specifications for patients with hemoglobinopathies: a systematic review and guideline’. Br J Haematol.

[13] Brasil. Ministério da Saúde (2015). Guia para o uso de hemocomponentes.

[14] Biller E, Zhao Y, Berg M, Boggio L, Capocelli KE, Fang DC (2018). Red blood cell exchange in patients with sickle cell disease-indications and management: a review and consensus report by the therapeutic apheresis subsection of the AABB. Transfusion.

[15] Estcourt LJ, Kohli R, Hopewell S, Trivella M, Wang WC (2020). Blood transfusion for preventing primary and secondary stroke in people with sickle cell disease. Cochrane Database Syst Rev.

[16] Estcourt LJ, Kimber C, Trivella M, Doree C, Hopewell S (2020). Preoperative blood transfusions for sickle cell disease. Cochrane Database Syst Rev.

[17] Estcourt LJ, Kimber C, Hopewell S, Trivella M, Doree C, Abboud MR (2020). Interventions for preventing silent cerebral infarcts in people with sickle cell disease. Cochrane Database Syst Rev.

[18] Lovett PB, Sule HP, Lopez BL (2017). Sickle cell disease in the emergency department. Hematol Oncol Clin North Am.

[19] Novelli EM, Gladwin MT (2016). Crises in sickle cell disease. Chest.

[20] Kozanoglu I, Ozdogu H (2018). Use of red blood cell exchange for treating acute complications of sickle cell disease. Transfus Apher Sci.

[21] Uda M, Naito S, Yamamoto K, Ishii A, Nishizaki T (1984). Optimal protocol for preparation of leukocyte-poor red cells with a blood cell processor. Transfusion.

[22] Baía F, Correia F, Alves B, Martinez F, Koch C, Carneiro A (2016). Phenotyping Rh/Kell and risk of alloimmunization in haematological patients. Transfus Med.

[23] Fasano RM, Miller MJ, Chonat S, Stowell SR (2019). Clinical presentation of delayed hemolytic transfusion reactions and hyperhemolysis in sickle cell disease. Transfus Clin Biol.

[24] Halford B, Pinheiro A, Haspel RL (2021). Hospital medicine providers’ transfusion knowledge: a survey study. Transfus Med Rev.

[25] Vaena MMV, Alves LA (2019). Assessment of the knowledge and perceptions of Brazilian medical residents on transfusion medicine. Hematol Transfus Cell Ther.

